# Intravascular large B‐cell lymphoma affecting multiple cranial nerves: A histopathological study

**DOI:** 10.1111/neup.12767

**Published:** 2021-09-19

**Authors:** Andrea Porzionato, Guido Pelletti, Luisa Barzon, Martina Contran, Aron Emmi, Angelo Arminio, Veronica Macchi, Raffaele De Caro

**Affiliations:** ^1^ Section of Human Anatomy, Department of Neuroscience University of Padova Padova Italy; ^2^ Section of Legal Medicine, Department of Medical and Surgical Sciences University of Bologna Bologna Italy; ^3^ Department of Molecular Medicine University of Padova Padova Italy

**Keywords:** cranial nerves, intravascular large B‐cell lymphoma, nerve palsy, neurolymphomatosis

## Abstract

Intravascular large B‐cell lymphoma (IVLBCL) is a rare form of lymphomas with poor prognosis, characterized by atypical lymphocytes selectively growing within the lumen of small or medium‐sized vessels. Here, we report a case of intracerebral IVLBCL in a 54‐year‐old man who died three months after symptom onset. The diagnosis was made by postmortem pathological examination, based on the identification of multiple ischemic lesions, with small or medium‐sized vessels filled with malignant B‐cells, in the cerebral hemispheres, cerebellum, midbrain, and medulla oblongata, including the external cuneate nucleus and trigeminal spinal tract nucleus. Apart from necrotic lesions, specific histopathological search for occluded vessels in the other brain stem structures permitted identification of significant involvement of the cuneate nucleus, solitary tract nucleus, hypoglossal nucleus, and inferior olivary complex. Small vessels affected by IVLBCL were also found in the trunks of the oculomotor, trigeminal, glossopharyngeal, vagal, and hypoglossal nerves. These histopathological findings were consistent with some cranial nerve symptoms/signs ascertained during hospitalization, such as diplopia, dysphonia, and asymmetry/hypomotility of the palatal veil. The case study presented here reports novel insights on radiological, anatomical, and clinical correlations of the IVLBCL, including the possible involvement of nuclei and trunks of multiple cranial nerves. The reported findings may help clinicians in the early identification of this rapidly progressive disease that can be easily misdiagnosed, through integrated neuroradiological, neurological and neuropathological approaches.

## INTRODUCTION

Intravascular large B‐cell lymphoma (IVLBCL), also known as intravascular lymphomatosis or angiotropic lymphoma, is a rare large B‐cell lymphoma characterized by atypical lymphocytes selectively growing within the lumen of small‐ or medium‐sized vessels. Inhibition of cell migration is probably involved in the pathogenesis, due to the lack of some molecules critical for extravasation, such as CD29.^1^, ^2^, ^3^ The incidence of this lymphoma is less than 1 per million.^4^, ^5^ The median age of patients is 70 years, ranging from 34 to 90 years, with the same frequency between males and females.^3^, ^6^, ^7^

From a histopathological point of view, intraluminal neoplastic cells are large and show a high nuclear/cytoplasmic ratio; nucleoli may be single or multiple and prominent. Lymphoma cells are highly proliferative, as shown by frequent mitotic figures and high Ki‐67 indices on immunohistochemistry. They express B‐cell markers, such as CD20, CD79a, and Pax‐5, but not a T‐cell marker CD3 or a macrophage marker CD68.^8^

IVLBCL may affect various tissues, organs, or both. Two main patterns of localization and clinical presentation have been identified as follows; one is the Western variant, showing a high frequency of skin and central nervous system involvement, and the other is the Asian variant, also called hemophagocytic syndromeassociated variant, displaying bone marrow involvement, thrombocytopenia, and hepatosplenomegaly.

The main pathogenic mechanism of nervous system damage is the occlusion of small vessels with consequent ischemic injury of the related regions. The histopathology of the central nervous system is characterized by appearance of multiple small infarcts associated with small or medium‐sized vessels filled with intraluminal noncohesive large cells in the ischemic lesions or at their borders. Leptomeningeal vessels may also be involved.

Brain magnetic resonance imaging (MRI) reveals normal findings or nonspecific hyperintense lesions in the white matter, suggestive of demyelination or ischemic pathology by small vessel occlusion.^8^, ^9^, ^10^

Laboratory tests are usually nonspecific. Malignant lymphoid cells are only occasionally found in the peripheral blood smear or in the cerebrospinal fluid, where an increased protein level and/or lymphocytosis are more common although unspecific.^8^, ^10^

Intra vitam diagnosis is particularly difficult due to the rapid course of the disease, the lack of involvement of lymph nodes and bone marrow, and the unspecific clinical, laboratory and imaging findings. Biopsies are needed to reach a diagnosis, but their success depends on lymphoma localization. In the classical Western variant, multiple cutaneous biopsies usually permit a diagnosis. When the central nervous system is selectively involved, brain biopsies are necessary, but even this invasive procedure may give false negative results if the lesions are not correctly targeted by sampling.

Treatment of IVLBCL is based on systemic chemotherapy, but it is generally ineffective due to aggressive behavior of neoplastic cells and rapid course of the disease. In some cases, high‐dose corticosteroids may provide transient clinical improvement. High‐dose chemotherapy with autologous cell transplantation has been proposed for young patients with an unfavorable prognosis.^8^

**Fig 1:**
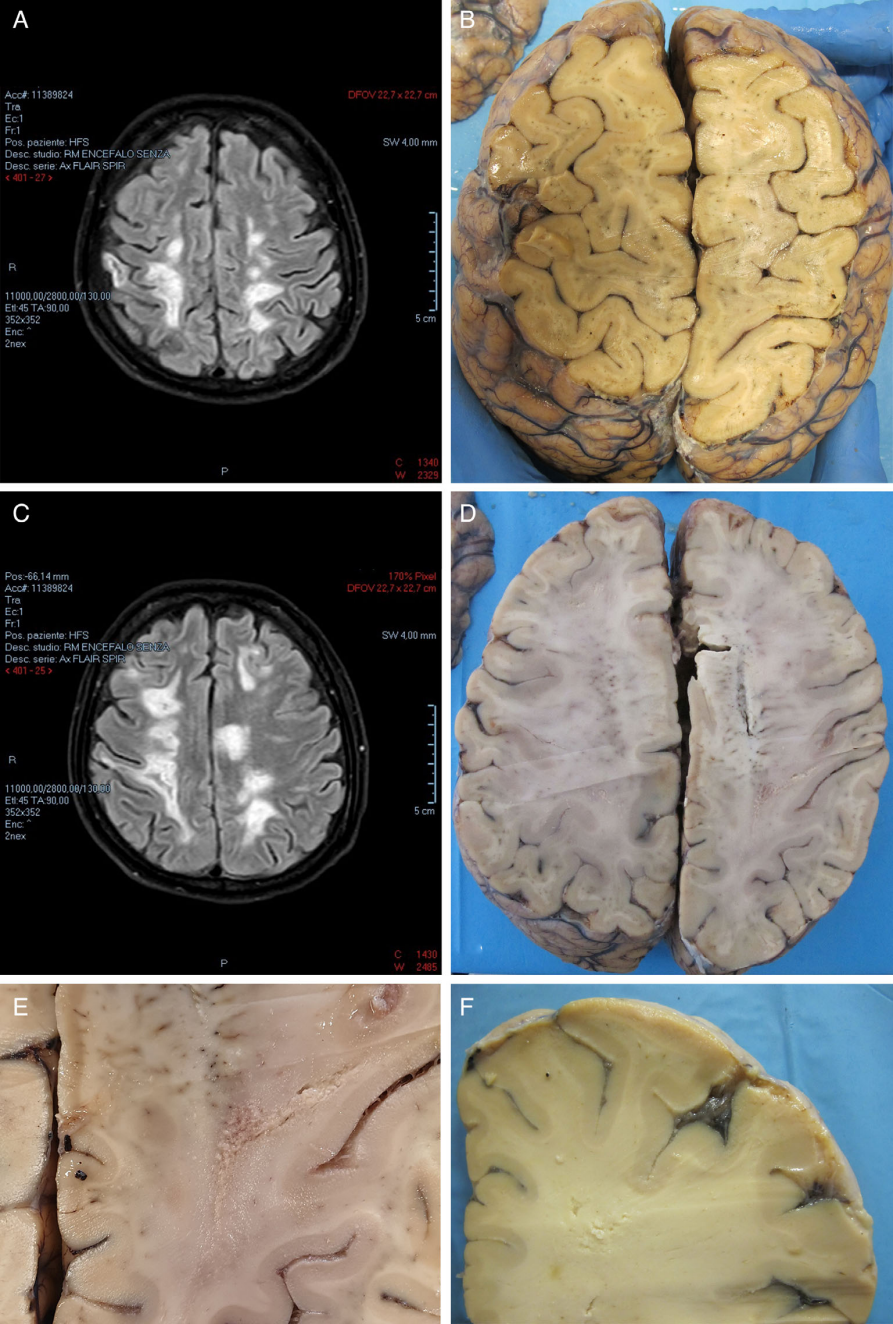
MRI (A, C) and gross (B, D‐F) findings of the cerebral hemispheres. (A, C) Axial FLAIR images taken on day 88 after first hospital admission show hyperintense areas in the cerebral white matter. (B, D) Horizontal brain slices corresponding to the imaging views (A, C) show the appearance of white matter lesions. (E, F) Details of lesions at higher magnifications are shown. Panels E and F correspond to selected area of slice D and taken at another slice.

**Fig 2:**
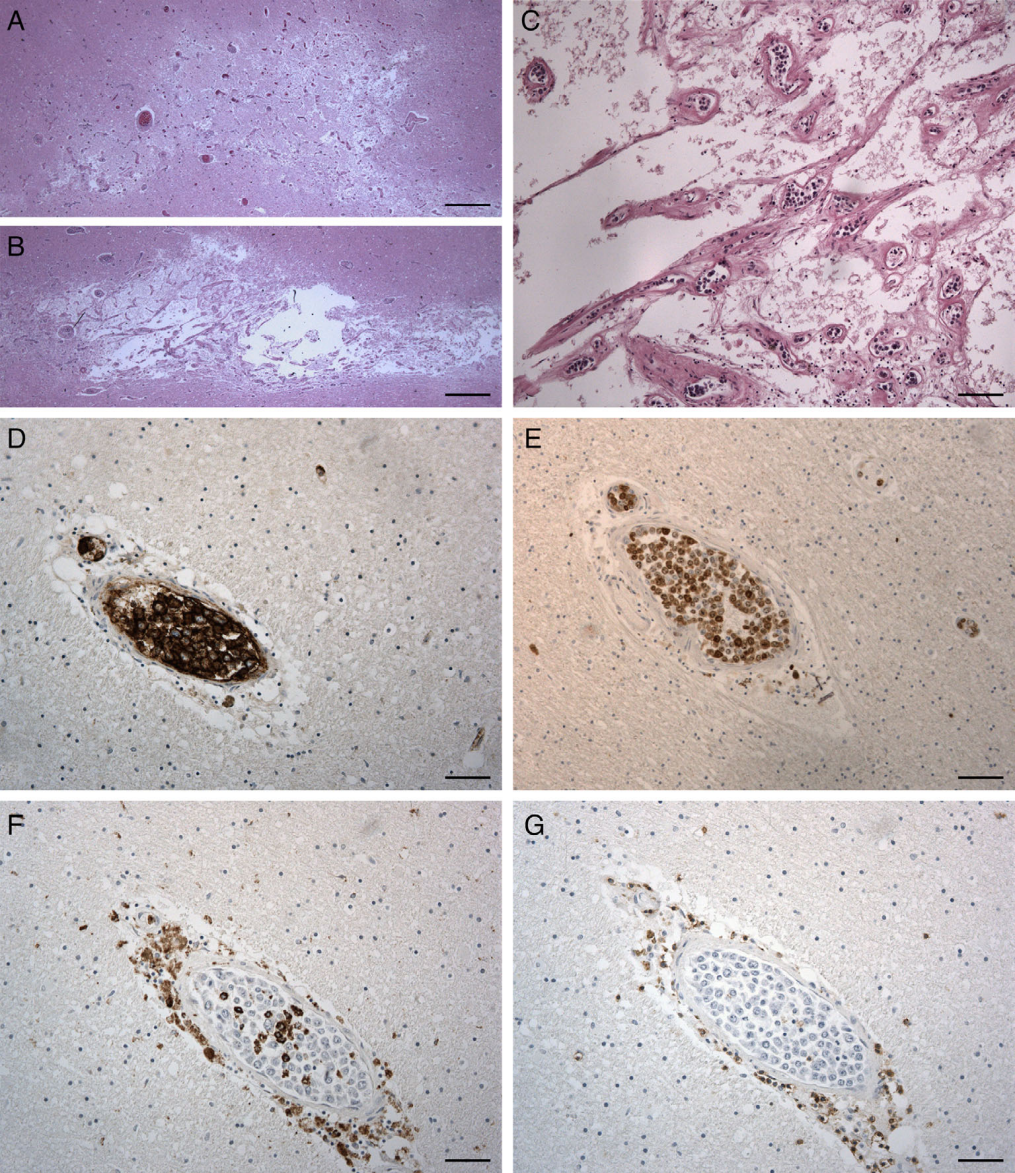
Microscopic findings of the cerebrum on HE staining (A‐C) and immunohistochemistry (D‐G). (A, B) Necrotic areas are observed in the hemispheric white matter. (C) At a higher magnification of panel (B), multiple small vessels with lumens filled by large round cells are observed. (D‐G) Almost all the intraluminal cells are positive for the B lymphocyte marker CD20 (D) and the proliferative ability marker Ki‐67 (E). Some intraluminal cells, positive for CD68, a marker of monocytes/macrophages/microglia, are observed (F). All the intraluminal cells are negative for the T lymphocyte marker CD3 (G). Note perivascular CD68‐positive or CD3‐positive cell infiltrates. Scale bars, 800 μm (A, B), 50 μm (D, F‐G), 100 μm (C, E).

**Fig 3:**
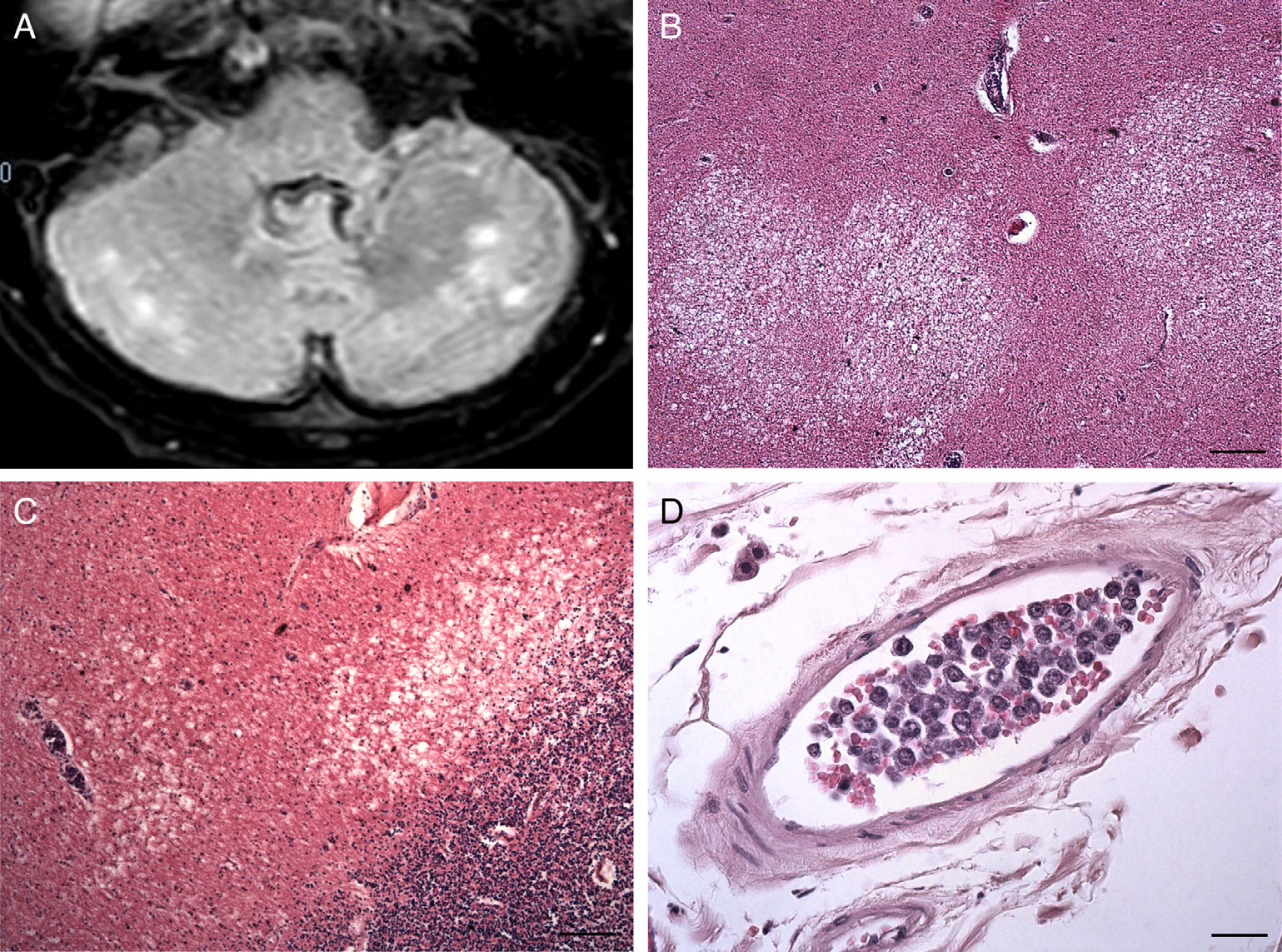
MRI (A) and histological (B‐D) findings of the cerebellum. (A) An axial FLAIR Spir image taken on day 88 after admission shows hyperintense lesions in the cerebellar white matter. (B, C) Necrotic lesions are observed in the cerebellar white matter. (D) A leptomeningeal medium‐sized vein is filled with large round cells. Scale bars, 200 μ (B), 100 μm (C), 25 μm (D).

**Fig 4:**
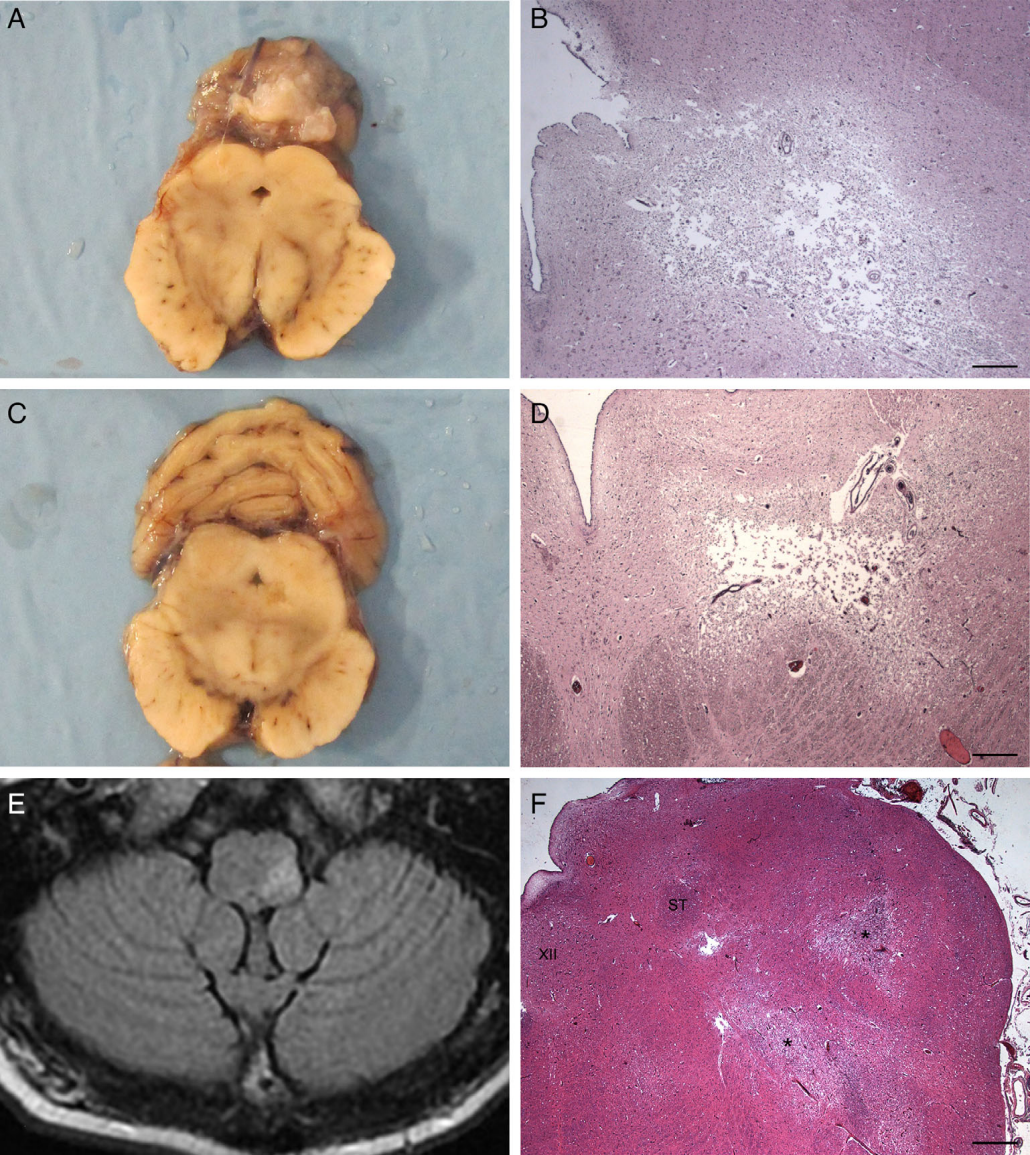
Gross (A, C), HE staining (B, D, F), and MRI (E) findings of the brain stem. (A) A transverse slice of the midbrain is shown. (B) A necrotic lesion is present on the left ventrolateral aspect of the midbrain aqueduct in the same level of (A). (C) Another transverse slice of the midbrain at the level caudal to (A) is shown. (D) A necrotic lesion is present on the left ventrolateral aspect of the midbrain aqueduct in the same level of (C). (E) An axial T2/FLAIR image taken on day 56 after admission shows a hyperintense lesion in the left posterolateral portion of medulla oblongata. (F) Necrotic lesions (asterisks) are also visible at the level of the left tegmentum of the medulla oblongata, including the trigeminal spinal tract nucleus and external cuneate nucleus. XII, hypoglossal nucleus; ST, solitary tract. Scale bars, 400 μm (B, D), 800 μm (F).

**Fig 5:**
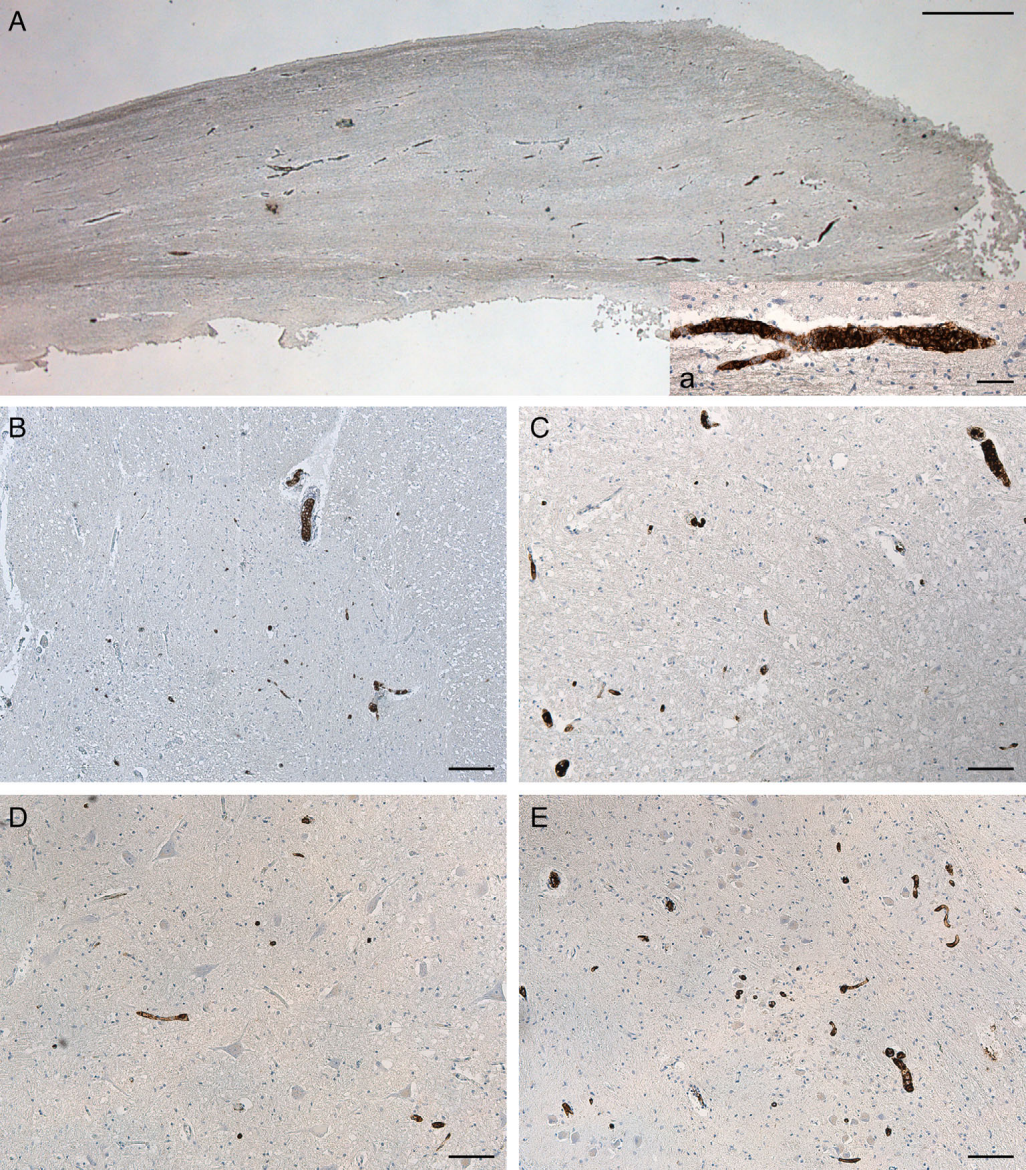
Immunohistochemical findings for CD20 in the olfactory bulb (A) and brain stem (B‐E). Vessels occluded by malignant B cells are present in the left olfactory bulb (A), left cuneate nucleus (B), left solitary tract nucleus (C), right hypoglossal nucleus (D), and left dorsal accessory olivary nucleus (E). Scale bars, 800 μm (A), 50 μμm (a), 200 μm (B), 100 μm (C‐E).

**Fig 6:**
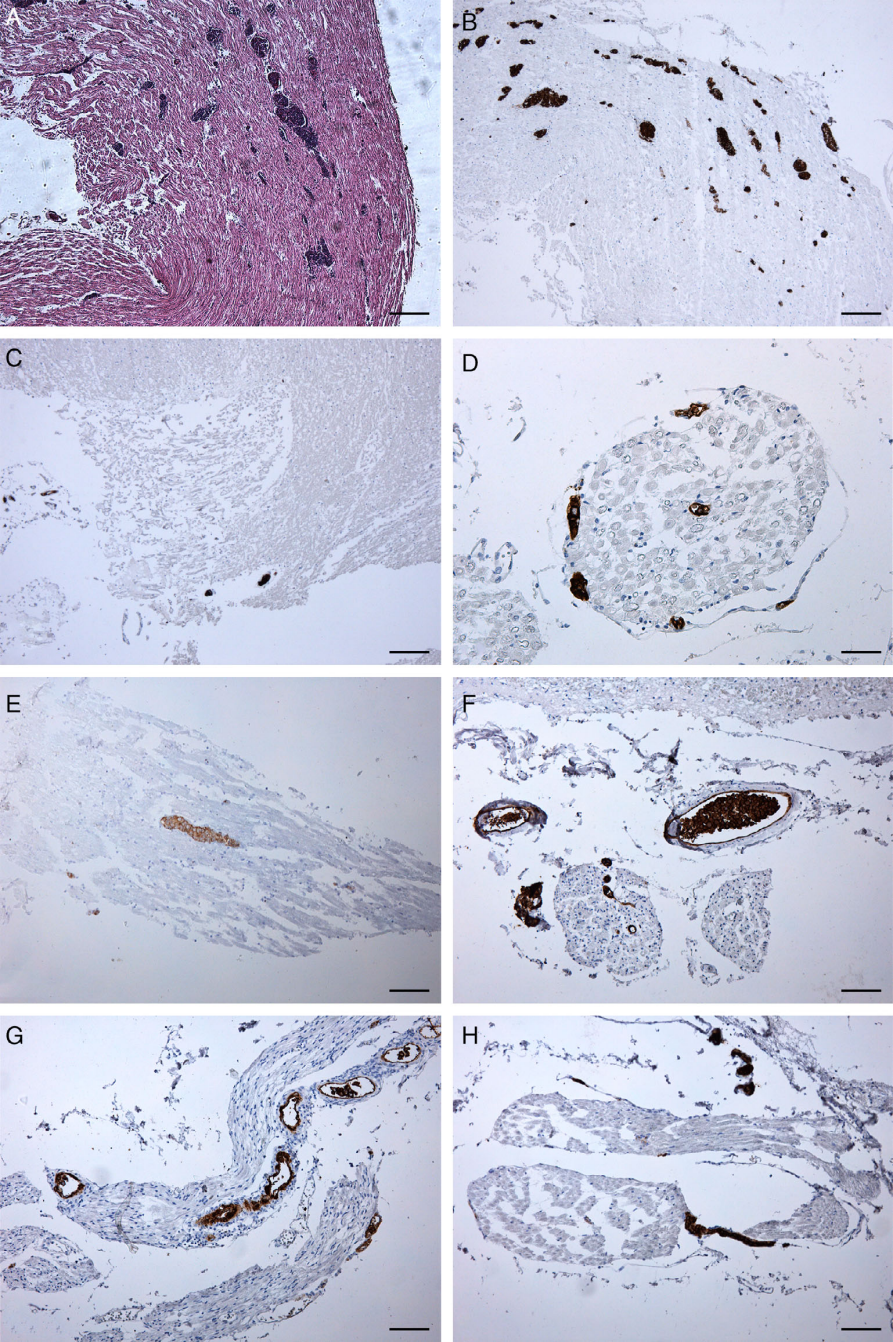
Findings of the cranial nerves on HE staining (A) and CD20 immunohistochemistry (B‐H). (A) Small vessels occluded by round cells are observed in the right oculomotor nerve trunk. (B‐H) Vessels occluded by CD20‐positive malignant B cells are visible in the right oculomotor (B), left (C, D) and right (E) trigeminal, left glossopharyngeal (F), right vagal (G), and right hypoglossal (H) nerves. Scale bars, 200 μm (A‐C), 50 μm (D), 100 μm (E‐H).

In the present report, we describe the neuropathological investigation (Figs. 1, 2, 3, 4, 5, 6) of a case of rapidly progressive IVLBCL with selective involvement of the brain and multiple cranial nerves, misdiagnosed in life as progressive multifocal leukoencephalopathy (PML). This is the first histopathological study demonstrating vessels occluded by malignant B‐cells in the nuclei and trunks of multiple cranial nerves.

Moreover, the patient was in therapy with tacrolimus for liver transplantation and, to the best of our knowledge, this case may be considered as the first case of post‐transplant lymphoproliferative disorder of IVLBCL type.

## CLINICAL SUMMARY

A 54‐year‐old man was admitted to a hospital with a history of eight weeks headache with slow and constant worsening of neurological deficits, which started as left paresthesia and evolved into left sensory and motor deficits with gait disorder and urinary incontinence. Ten months before admission, the patient underwent liver transplantation for hepatitis C virus‐associated cirrhosis complicated by hepatocellular carcinoma. After transplantation, immunosuppressive therapy with tacrolimus was started.

On the first day of hospitalization, computed tomography (CT) and MRI of the brain revealed lesions in the right temporoparietal lobes and the left temporoparietal and frontal lobes. ^18^Fluorodeoxyglucose‐positron emission tomography (^18^FDG‐PET) documented hyperfixation of the ^18^FDG at the left temporoparietal site, as well as tenuous and diffuse hyperfixation at medulla oblongata with nonunivocal interpretation.

Microbiological investigations included blood cultures, galactomannans testing in serum, interferon gamma release assay for tuberculosis diagnosis, molecular testing in blood samples and cerebrospinal fluid (CSF) and serology testing for John Cunningham virus (JCV), Epstein–Barr virus (EBV), West Nile virus, Usutu virus, Toscana virus, *Cryptococcus neoformans*, *Toxoplasma gondii*, *Leishmania* spp., *Tropheryma whippelii*, *Borrelia* spp., *Brucella* spp., *Salmonella* spp., *Mycoplasma pneumoniae*, *Coxiella burnetii*, *Chlamydophila pneumoniae*, and *Legionella pneumophila*. The results of all these tests were negative.

On day 30, the patient showed hypomobility, spasticity, dysreflexia, exhaustible clonus, and presence of Babinski's sign. He was transferred to the intensive care unit. Virological testing on CSF was repeated, and the results were negative. Nonetheless, PML was suspected. Hence, immunosuppressive therapy was modified with suspension of tacrolimus and introduction of low doses of everolimus.

On day 40, a repeated brain MRI identified necrotic cavitation of the largest lesion in the right Rolandic region. Neurosurgeons advised against the possibility of a brain biopsy due to the location of the major lesions and the coexistence of thrombocytopenia.

On day 56, brain MRI revealed the appearance of a hyperintense lesion on T2‐weighted and fluid‐attenuated inversion recovery (FLAIR) images of the left posterolateral portion of the medulla oblongata, as well as a slight restriction of the signal on diffusion‐weighted images (DWIs) (Fig. 4E). A slight increase in size of the lesions previously detected was observed, with necrotic cavitations in some, in particular at the left hemisplenium of the corpus callosum, in the left paratrigonal area and in the right centrum semiovale.

On day 64, diplopia and a further neurological deterioration were observed. Immunosuppressive therapy was indefinitely suspended, and the patient was discharged home.

After initially being clinically stable at home, on day 87, the patient was readmitted to the hospital with rapid neurological deterioration associated with fever. The patient was alert and reactive with normal vital parameters. Neurological examination revealed fluent speech with slight dysphonia, slight left facial palsy, and asymmetry of the palatal veil, with right hypomotility. Hypotonia and hyposthenia of the lower limbs, left hemiparesis, dysreflexia, the presence of Babisnki's sign, and right hemineglect were also observed.

On day 88, brain MRI revealed an increase in the size of the hyperintense lesions previously described on day 56, with a signal restriction on DWIs (Fig. 1A, C). In addition, two further gross lesions were detected in the body and splenium of the corpus callosum, with a slight mass effect on adjacent ventricular structures and additional multiple lesions present in the right temporal white matter, both sides of the cerebellar hemispheres, and left middle cerebellar peduncle.

In the hypothesis of a PML with negative virological test results, brain biopsy was planned, but it was not executed due to rapid worsening followed by death of the patient on day 91.

## PATHOLOGICAL FINDINGS

Autopsy was performed two days after death of the patient. External examination and cadaveric sections were negative, without pathological findings of the skin, heart, lungs, liver, kidneys, and other organs.

CSF and small brain samples from the temporal lobes were taken and frozen for molecular testing of infectious agents.

Macroscopic examination of the brain did not reveal any surface alteration. The vessels of the cerebral base did not show anatomical variations or anomalies. The brainstem was separated through a transverse slices at the level of the midbrain. The cerebrum was sliced through horizontal planes. Multiple malacic areas were observed in the subcortical white matter, characterized by reduced consistency and tissue destruction, with spongiotic appearance and cavitation (Fig. 1). The cortex and deep gray matter were normal.

The brain stem and cerebellum were cut together into 15 slices perpendicular to the brain stem axis. The cerebellar white matter including necrotic lesions showed some areas of reduced consistence and discromy (Fig. 3). The cerebellar cortex and nuclei appeared normal. A necrotic dyschromic area extending from the left anterior slope of the mesencephalic aqueduct was observed (Fig. 4). Small dyschromic areas were also visible in the medullary tegmentum. The brain slices were fixed in 10% formalin and embedded in paraffin, followed by thin sectioning. Paraffin‐embedded sections were stained with hematoxylin and eosin (HE) and processed for immunohistochemistry.

On histopathological examination, the above lesions showed necrosis and tissue destruction, without blurred hemorrhage (Fig. 2). In the context of each necrotic lesion, or at its immediate borders, small or middle‐sized vessels were almost completely filled with loosely packed round cells having large pleomophic nuclei, prominent central nucleoli, and scanty cytoplasm (Figs. 2, 3) while no extravascular cell infiltrates were detected in the brain parenchyma. Immunohistochemical analyses were performed with the following antibodies and protocols: a rabbit monoclonal anti‐human Ki‐67 antibody (clone SP6; Spring Bioscience Corporation, San Francisco, US; Ref. M3062; heat pretreatment with high pH ethylene diamine tetraacetic acid (EDTA) in Tris buffer for antigen retrieval; 1:200), a rabbit polyclonal anti‐human CD3 antibody (Dako, Glostrup, Denmark; Ref. GA503; heat pretreatment with low pH citrate buffer for antigen retrieval; 1:500), and a mouse monoclonal anti‐human CD68 antibody (clone KP‐1, Dako; Ref. M0814; heat pretreatment with high pH EDTA buffer for antigen retrieval; 1:5000). Antigen retrieval was performed on a Dako EnVision Station water bath according to the manufacturer's recommendations.

Almost all luminal cells were positive for CD20 and negative for CD3. Most cells were positive for Ki‐67, a proliferative ability marker. Isolated cells were positive for CD68, a macrophage/microglia lineage cell (Fig. 2). Some leptomeningeal small or medium‐sized vessels also showed intraluminal occlusion by lymphoid cells displaying the abovementioned characteristics. Thus, the histological and immunohistochemical characteristics of the cells were consistent with a diagnosis of IVLBCL. In some vessels, apart from the above large cells in the lumen, some perivascular CD68‐positive macrophage/microglia and CD3‐positive small lymphocytes were also present.

In the medulla oblongata, ischaemic necrotic lesions were found in the left tegmentum, including the external cuneate nucleus and trigeminal spinal tract nucleus. A thorough histopathological analysis was also performed on all the brain stem regions by CD20 immunohistochemistry to evaluate the localization of small vessels occluded by IVLBC cells in other structures not yet clearly damaged. This further analysis permitted identification of small vessels occluded by CD20‐positive malignant B cells in the context of left cuneate nucleus, left solitary tract nucleus, right hypoglossal nucleus, and left inferior olivary complex, particularly at the level of the dorsal accessory olivary nucleus (Fig. 5).

Trunks of cranial nerves included in the brain stem samples were also specifically evaluated. In particular, small vessels in the trunks of the right oculomotor, both sides trigeminal, left glossopharyngeal, right vagal, and right hypoglossal nerves showed occlusion by atypical round cells with the above morphological characteristics (Fig. 6). The right oculomotor nerve showed a higher number of vessels involved. Unfortunately, it was not possible to evaluate cranial nerves (I, II, IV, VI, VII, VIII) because they were not originally included in the samples. The olfactory bulbs and tracts also showed many vessels containing malignant B‐cells (Fig. 5A).

Histopathological analysis of the other non‐neural tissues, such as heart, liver, pancreas, lung, kidneys, adrenal glands, lymph nodes, did not show IVBCL cell‐occluded vessels. Skin samples were not available.

Real‐time polymerase chain reaction analysis performed on samples of brain lesions were negative for genome sequences of JCV, herpes simplex virus (HSV), varicella zoster virus (VZV), EBV, cytomegalovirus (CMV), human herpes virus 6 (HHV‐6), and HHV‐8.

## DISCUSSION

In this study, we report a case of IVLBCL with rapidly progressive neurological symptoms and extensive involvement of the cerebrum, cerebellum, brainstem and cranial nerves.

Anamnestic, clinical, and neuroimaging data initially pointed toward a PML, which was considered the most likely underlying disease until death occurred. Even, the patient had undergone liver transplantation and was under treatment with the immunosuppressor tacrolimus, which is a well‐known predisposing factor for PML.^11^ Although testing for JCV DNA in CSF was repeatedly negative, the main diagnostic hypothesis remained unchanged. In fact, the negativity of the CSF to JCV test is not an uncommon finding in PML cases.^11^, ^12^, ^13^, ^14^

Conversely, although this is the first report of IVLBCL in a post‐transplantation patient, we must consider that long‐term immunosuppression after solid organ transplantation places recipients at an increased risk of post‐transplantation lymphoproliferative disorders (PTLD).^15^ Komeno *et al*.^16^ also reported IVLBCL in a patient, affected by rheumatoid arthritis, was treated with tacrolimus and methotrexate. PTLD is mostly of B‐cell origin and shows rapid onset, aggressive behavior, and a predilection for extranodal sites. Partial or complete regression after reduction or withdrawal of immunosupressant doses for therapy has been previously reported.^15^ Thus, on the basis of the above data, a potential role of long‐term immunosuppression may be hypothesized in the pathogenesis and rapid progression of the disease in the present case.

Diagnosis of IVLBCL is a major challenge as clinical signs and patterns of MRI and PET are nonspecific.^17^, ^18^ The only way to make a diagnosis before death is to perform a brain biopsy which is a highly invasive procedure not free from possible false negative results.^19^ As a consequence, the risk–benefit ratio must be carefully evaluated case by case. In the present case, as often reported in the literature,^17^, ^18^, ^19^ the correct diagnosis was made by postmortem pathological examination, because the patient died before biopsy was performed.

A multidisciplinary postmortem study of the case, integrated with antemortem data, allowed the identification of novel neuropathological features at the level of the medulla oblongata and cranial nerves.

In the literature, there have been various MRI reports of brain stem necrotic lesions in IVLBCL, with the medulla oblongata as a part being the most rarely affected.^20^, ^21^ In the present case, necrotic lesions were well‐documented on neuroimaging at the level of the midbrain and medulla oblongata. Nevertheless, a detailed histopathological analysis was performed on all the brain stem structures in order to address the presence of occluded vessels also in non‐necrotic structures; this may explain some of the clinical manifestations. In particular, in the medulla oblongata, we found multiple vessels occluded by lymphomatous cells in various nuclei, such as the gracilis nucleus, solitary tract nucleus, hypoglossal nucleus, and inferior olivary complex. Involved vessels were also sparsely present in the pons and midbrain.

Clinical signs and symptoms indicating cranial nerve involvement have been quite frequently reported, including visual impairment and reduced conduction velocity of optic nerves, diplopia, extraocular muscle palsy, trigeminal hypaesthesia/dysaesthesia/pain or palsy, facial palsy, dizziness, hearing loss, dysarthria, and swallowing disturbance.^19^, ^20^, ^22^, ^23^, ^24^, ^25^, ^26^, ^27^, ^28^, ^29^, ^30^, ^31^, ^32^, ^33^, ^34^ However, in most cases, it was not clarified whether the brain stem cranial nerve nuclei or trunks were affected. Direct involvement of trunks of cranial nerves was demonstrated in only a couple of cases via results on neuroimaging or histopathology. In a patient with left facial hypoesthesia, high signal intensity of a thickened trigeminal nerve with T1‐weighted images on brain MRI, together with increased ^18^FDG‐uptake in the homolateral Gasserian ganglion. The patient was successfully treated with chemotherapy using ifosfamide, etoposide, cytarabine, and methotrexate, and the lesion in the trigeminal nerve was no longer visible on follow‐up MRI and PET.^29^, ^30^ Histopathological involvement of both the trigeminal nerves has been reported in an autopsy case, although images of the microscopic findings were not shown in the study.^27^

Interestingly, in the present case, IVLBCL‐involved vessels were found in the oculomotor, trigeminal, glossopharyngeal, vagal, and hypoglossal nerves as well as in the olfactory bulbs and tracts. Therefore, diplopia and paralysis of the palatine veil can be attributed to the involvement of the oculomotor nerve and glossopharyngeal or vagal nerve, respectively, and the observation could support the importance of brain stem and cranial nerve involvement for pathophysiological mechanisms and clinical implications of IVLBCL.

To the best of our knowledge, this is the first histopathological confirmation of involvement of central and peripheral components of the cranial nerves that could, at least in part, explain the neurological symptoms of the disease.

In conclusion, this report highlights novel insights on clinicoanatomical correlations in IVLBCL, including the involvement of central and peripheral components of a number of the cranial nerves. The reported findings may be useful for identification of this rapidly progressive disease through an integrated neurological, neuroradiological, and neuropathological approaches. From a methodological point of view, we suggest that on postmortem pathological examination, the cranial nerves should be specifically sampled before brain stem slicing in order to permit a systematic evaluation.

## DISCLOSURES

The authors declare no specific funding and no conflict of interest for this article.

## References

[1] Ferry JA , Harris NL , Picker LJ *et al*. Intravascular lymphomatosis (malignant angioendotheliomatosis). A B‐cell neoplasm expressing surface homing receptors. Mod Pathol 1988; 1: 444–452. 3065781

[2] Ponzoni M , Arrigoni G , Gould VE *et al*. Lack of CD 29 (beta1 integrin) and CD 54 (ICAM‐1) adhesion molecules in intravascular lymphomatosis. Hum Pathol 2000; 31: 220–226. 10685637 10.1016/s0046-8177(00)80223-3

[3] Ponzoni M , Campo E , Nakamura S . Intravascular large B‐cell lymphoma: A chameleon with multiple faces and many masks. Blood 2018; 132: 1561–1567. 30111607 10.1182/blood-2017-04-737445

[4] Orwat DE , Batalis NI . Intravascular large B‐cell lymphoma. Arch Pathol Lab Med 2012; 136: 333–338. 22372911 10.5858/arpa.2010-0747-RS

[5] Mansueto G , Di Vito A , Belluomo C *et al*. A case of intravascular large B cell lymphoma: New clinical and immunohistochemical findings. Neuropathology 2016; 36: 496–503. 27090763 10.1111/neup.12300

[6] Ferreri AJ , Campo E , Seymour JF *et al*. Intravascular lymphoma: Clinical presentation, natural history, management and prognostic factors in a series of 38 cases, with special emphasis on the ‘cutaneous variant’. Br J Haematol 2004; 127: 173–183. 15461623 10.1111/j.1365-2141.2004.05177.x

[7] Nakamura S , Ponzoni M , Campo E . Intravascular large B‐cell lymphoma. In: Swerdlow SH , Campo E , Harris NL *et al*., (eds). WHO Classification of Tumours of Haematopoietic and Lymphoid Tissue (IARC WHO Classification of Tumours). Lyon: IARC, 2008; 252–254.

[8] Mihaljevic B , Sternic N , Skender Gazibara M *et al*. Intravascular large B‐cell lymphoma of central nervous system ‐ a report of two cases and literature review. Clin Neuropathol 2010; 29: 233–238. 20569674 10.5414/npp29233

[9] Song DK , Boulis NM , McKeever PE , Quint DJ . Angiotropic large cell lymphoma with imaging characteristics of CNS vasculitis. AJNR Am J Neuroradiol 2002; 23: 239–242. 11847048 PMC7975247

[10] Kubisova K , Martanovic P , Sisovsky V *et al*. Dominant neurologic symptomatology in intravascular large B‐cell lymphoma. Bratisl Lek Listy 2016; 117: 308–311. 27546361 10.4149/bll_2016_061

[11] Aridon P , Ragonese P , Di Benedetto N *et al*. Progressive necrotic encephalopathy following tacrolimus therapy for liver transplantation. Neurol Sci 2009; 30: 527–529. 19779798 10.1007/s10072-009-0149-0

[12] Mazda ME , Brosch JR , Wiens AL *et al*. A case of natalizumab‐associated progressive multifocal leukoencephalopathy with repeated negative CSF JCV testing. Int J Neurosci 2013; 123: 353–357. 23252596 10.3109/00207454.2012.760561

[13] Lopes CCB , Crivillari M , Prado JCM *et al*. Progressive multifocal leukoencephalopathy: A challenging diagnosis established at autopsy. Autops Case Rep 2019; 9: e2018063. 30863734 10.4322/acr.2018.063 PMC6394363

[14] Lee SY , Ko HC , Kim SI , Lee YS , Son BC . Progressive multifocal leukoencephalopathy diagnosed by brain biopsy, not by the DNA test for JC virus. Asian J Neurosurg 2019; 14: 240–244. 30937044 10.4103/ajns.AJNS_243_17 PMC6417347

[15] Leblond V , Choquet S . Lymphoproliferative disorders after liver transplantation. J Hepatol 2004; 40: 728–735. 15094218 10.1016/j.jhep.2004.03.006

[16] Komeno Y , Akiyama M , Okochi Y *et al*. Hemophagocytic syndrome‐associated variant of methotrexate‐associated intravascular large B‐cell lymphoma in a rheumatoid arthritis patient. Case Rep Hematol 2019; 2019: 8947616. 31612088 10.1155/2019/8947616 PMC6755279

[17] Abe Y , Narita K , Kobayashi H *et al*. Clinical value of abnormal findings on brain magnetic resonance imaging in patients with intravascular large B‐cell lymphoma. Ann Hematol 2018; 97: 2345–2352. 30141061 10.1007/s00277-018-3481-8

[18] Barranco R , Caputo F , Bedocchi D *et al*. Unusual and fatal case of an undiagnosed intravascular large B‐cell lymphoma: The Oncologist's great imitator. J Forensic Sci 2020; 65: 314–317. 31361917 10.1111/1556-4029.14141

[19] Bhargava P , Siddiqui F , Aggarwal B , Moore BE , Elble RJ . A unique case of intravascular lymphoma mimicking encephalomyeloradiculoneuropathy. Neurologist 2015; 20: 18–21. 26185958 10.1097/NRL.0000000000000042

[20] Berger JR , Jones R , Wilson D . Intravascular lymphomatosis presenting with sudden hearing loss. J Neurol Sci 2005; 232: 105–109. 15850590 10.1016/j.jns.2005.01.001

[21] Simeni Njonnou SR , Couturier B , Gombeir Y *et al*. Pituitary gland and neurological involvement in a case of Hemophagocytic syndrome revealing an intravascular large B‐cell lymphoma. Case Rep Hematol 2019; 2019: 9625075. 31183225 10.1155/2019/9625075 PMC6512020

[22] Madara J , Shane J , Scarlato M . Systemic endotheliomatosis: A case report. J Clin Pathol 1975; 28: 476–482. 1141448 10.1136/jcp.28.6.476 PMC475746

[23] Yamamura Y , Akamizu H , Hirata T , Kito S , Hamada T . Malignant lymphoma presenting with neoplastic angioendotheliosis of the central nervous system. Clin Neuropathol 1983; 2: 62–68. 6851298

[24] Bhawan J , Wolff SM , Ucci AA , Bhan AK . Malignant lymphoma and malignant angioendotheliomatosis: One disease. Cancer 1985; 55: 570–576. 3880661 10.1002/1097-0142(19850201)55:3<570::aid-cncr2820550316>3.0.co;2-0

[25] Elner VM , Hidayat AA , Charles NC *et al*. Neoplastic angioendotheliomatosis. A variant of malignant lymphoma immunohistochemical and ultrastructural observations of three cases. Ophthalmology 1986; 93: 1237–1245. 3808635

[26] Stroup RM , Sheibani K , Moncada A , Purdy LJ , Battifora H . Angiotropic (intravascular) large cell lymphoma. A clinicopathologic study of seven cases with unique clinical presentations. Cancer 1990; 66: 1781–1788. 1698530 10.1002/1097-0142(19901015)66:8<1781::aid-cncr2820660824>3.0.co;2-5

[27] Glass J , Hochberg FH , Miller DC . Intravascular lymphomatosis. A systemic disease with neurologic manifestations. Cancer 1993; 71: 3156–3164. 8490846 10.1002/1097-0142(19930515)71:10<3156::aid-cncr2820711043>3.0.co;2-o

[28] Wake A , Kakinuma A , Mori N *et al*. Angiotropic lymphoma of paranasal sinuses with initial symptoms of oculomotor nerve palsy. Intern Med 1993; 32: 237–242. 8329819 10.2169/internalmedicine.32.237

[29] Koyama T , O'uchi T , Matsue K . Neurolymphomatosis involving the trigeminal nerve and deep peroneal nerve in a patient with relapsed intravascular large B‐cell lymphoma. Eur J Haematol 2010; 85: 275–276. 20528901 10.1111/j.1600-0609.2010.01477.x

[30] Matsue K , Hayama BY , Iwama K *et al*. High frequency of neurolymphomatosis as a relapse disease of intravascular large B‐cell lymphoma. Cancer 2011; 117: 4512–4521. 21448935 10.1002/cncr.26090

[31] Yoshida S , Kuroda H , Fukuhara N *et al*. Heparin‐responsive angiopathy in the central nervous system caused by intravascular large B‐cell lymphoma. J Neurol Sci 2015; 352: 117–119. 25829082 10.1016/j.jns.2015.03.026

[32] Sato S , Teshima S , Nakamura N *et al*. Intravascular large B‐cell lymphoma involving large blood vessels, three autopsy cases. Pathol Int 2019; 69: 97–103. 30672647 10.1111/pin.12751

[33] Miyake Z , Tomidokoro Y , Tsurubuchi T *et al*. Intravascular large B‐cell lymphoma presenting with hearing loss and dizziness: A case report. Medicine 2019; 98: e14470. 30762766 10.1097/MD.0000000000014470 PMC6407998

[34] Ong YC , Kao HW , Chuang WY *et al*. Intravascular large B‐cell lymphoma: A case series and review of literatures. Biomed J 2020: S2319‐4170(20)30041‐X. 10.1016/j.bj.2020.04.005 PMC8514799 32344119

